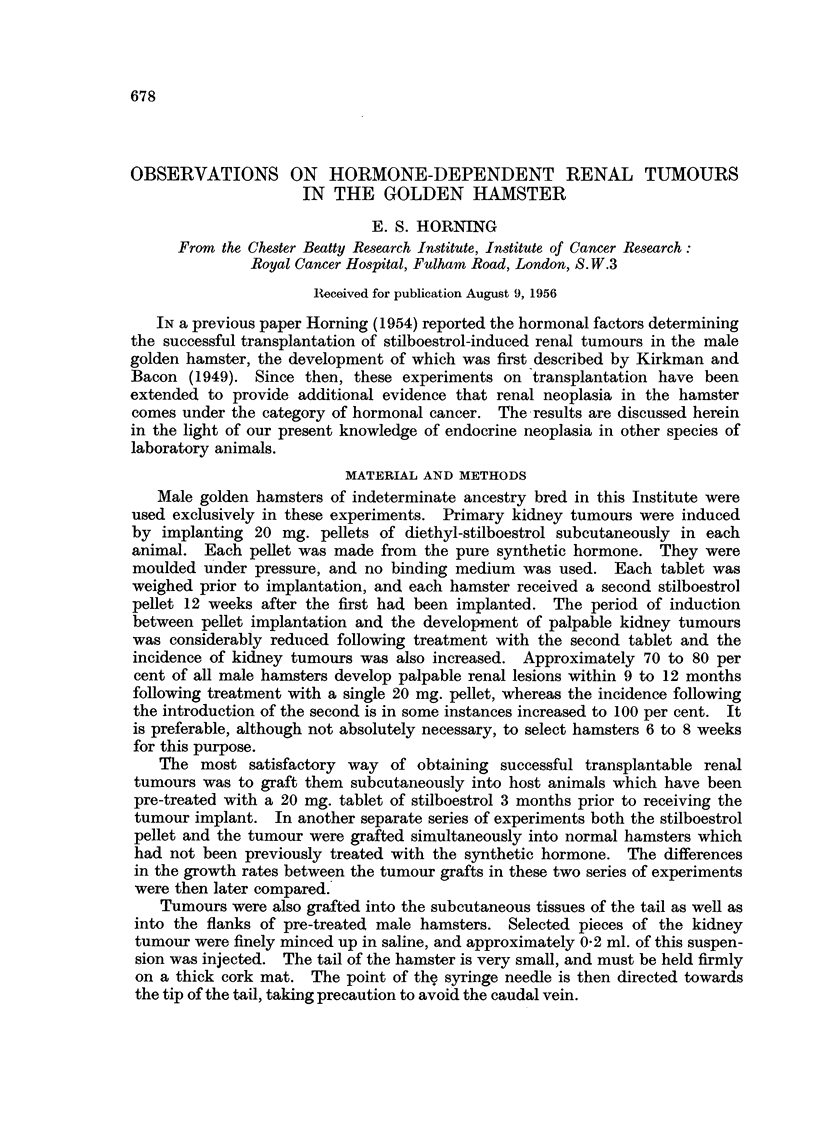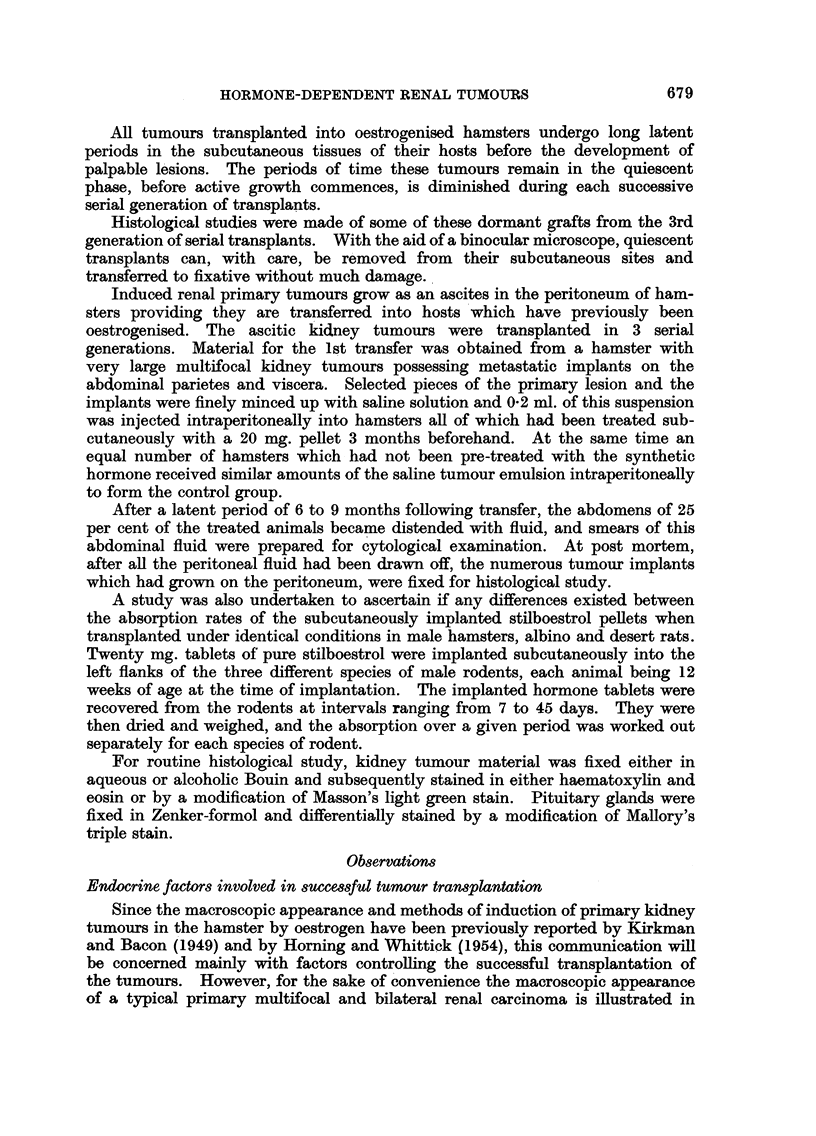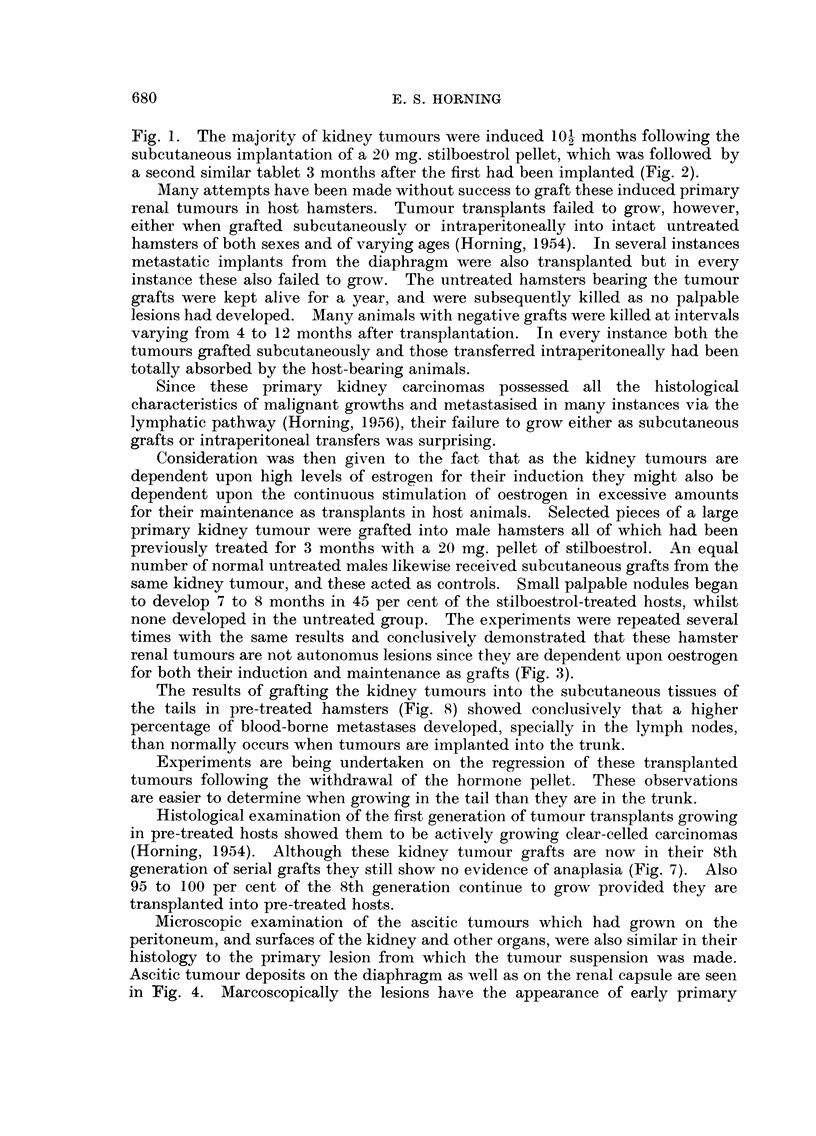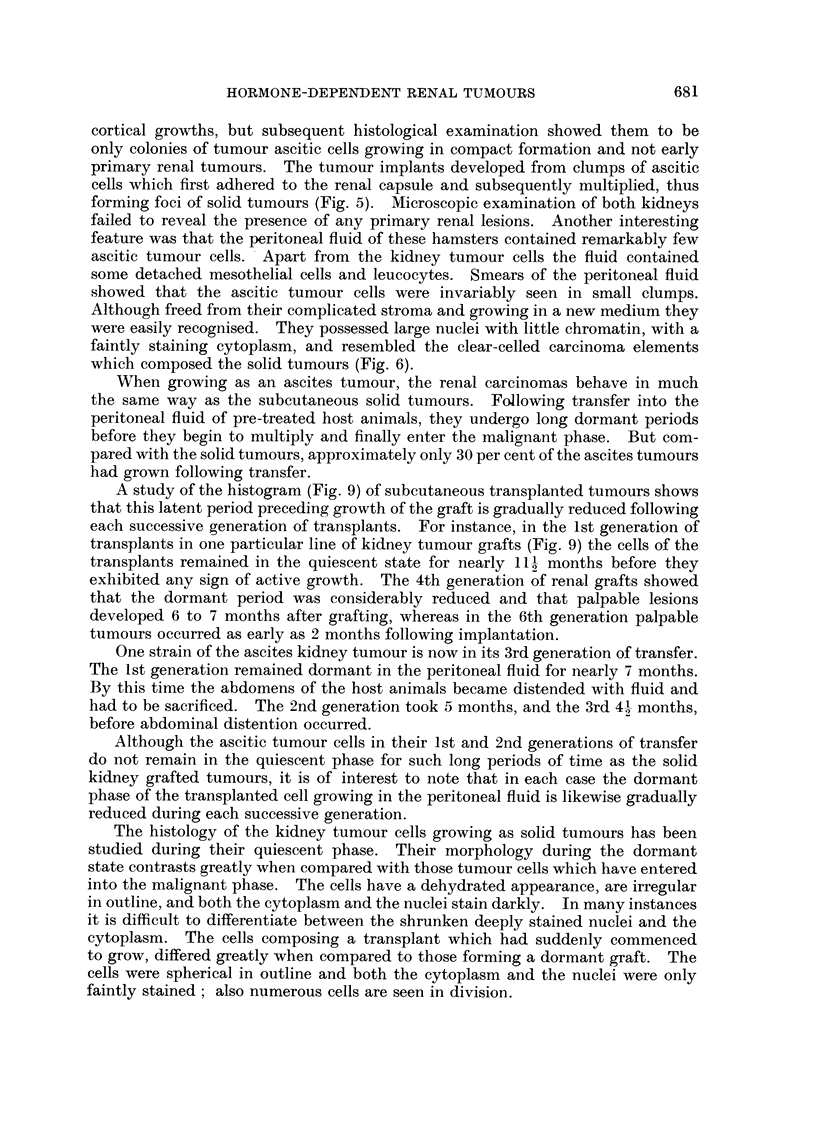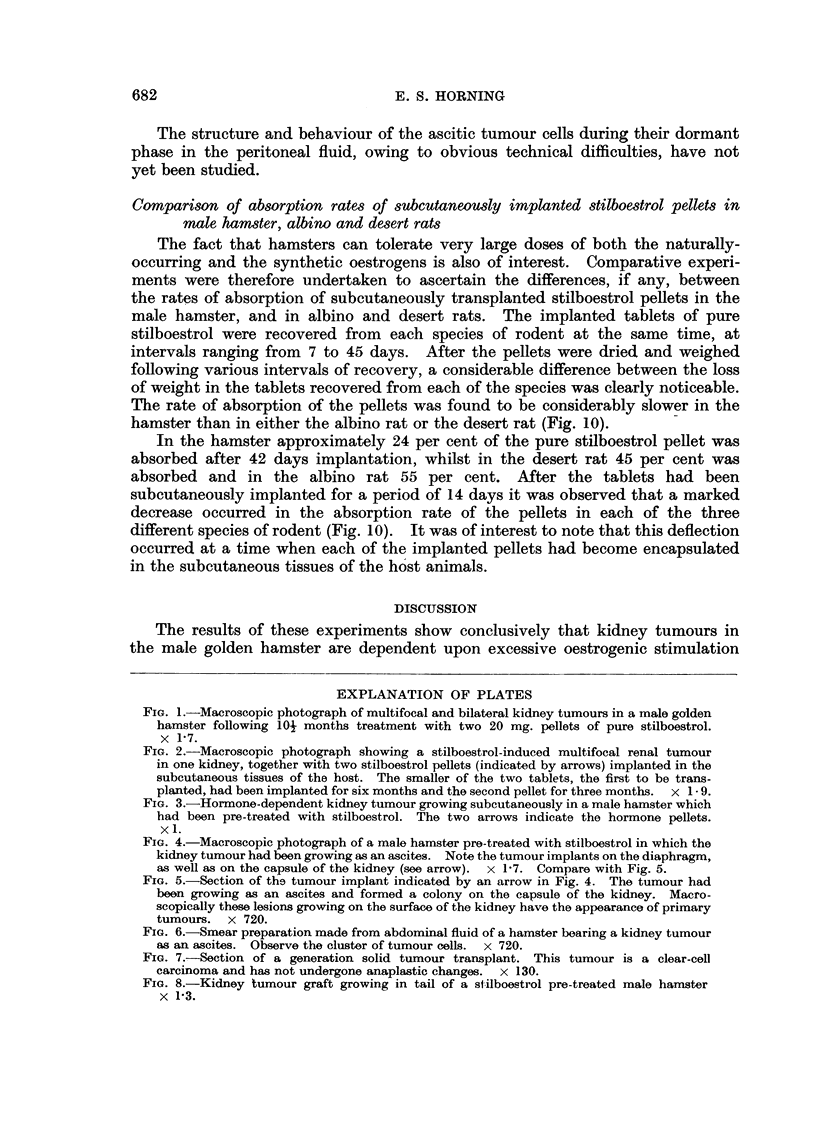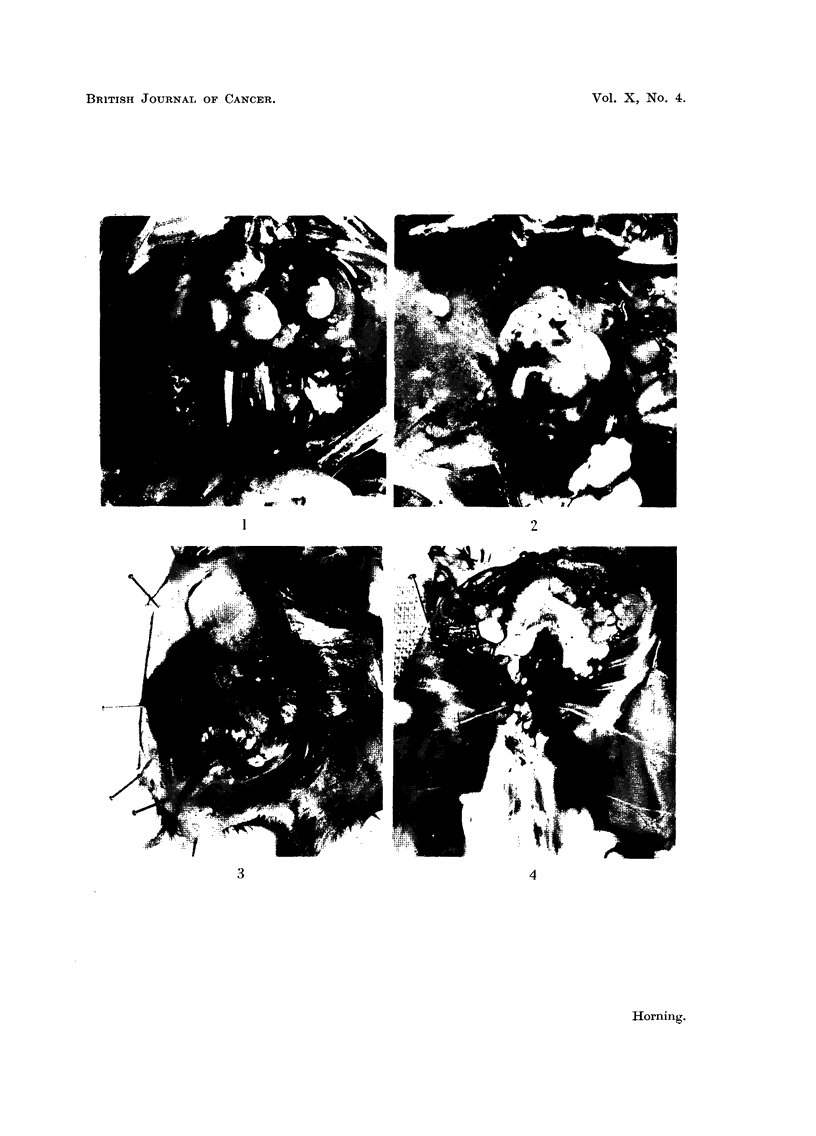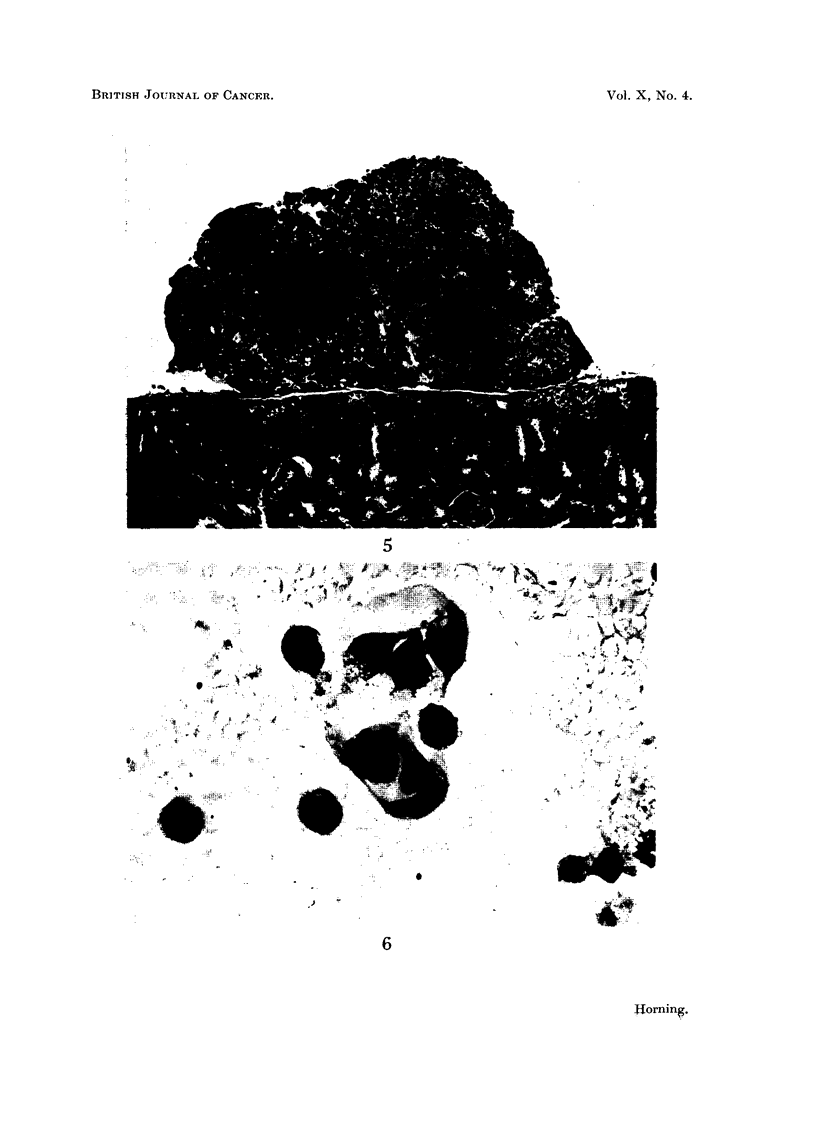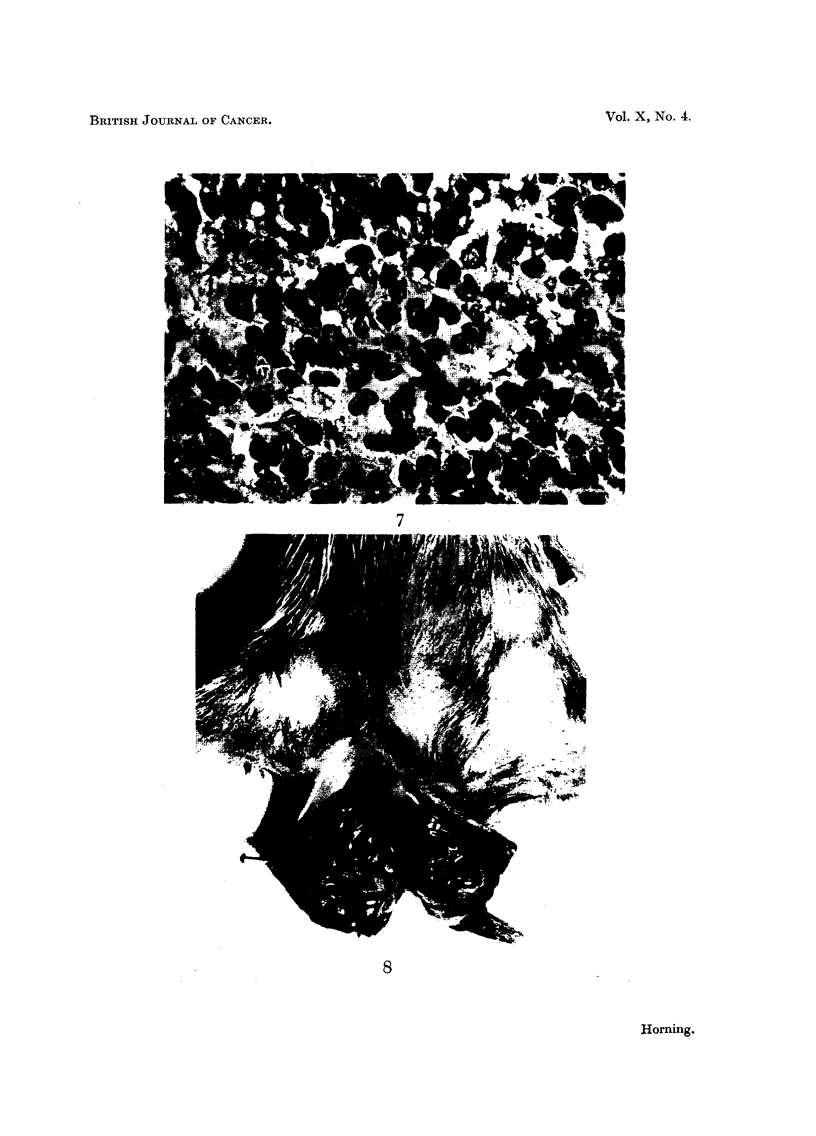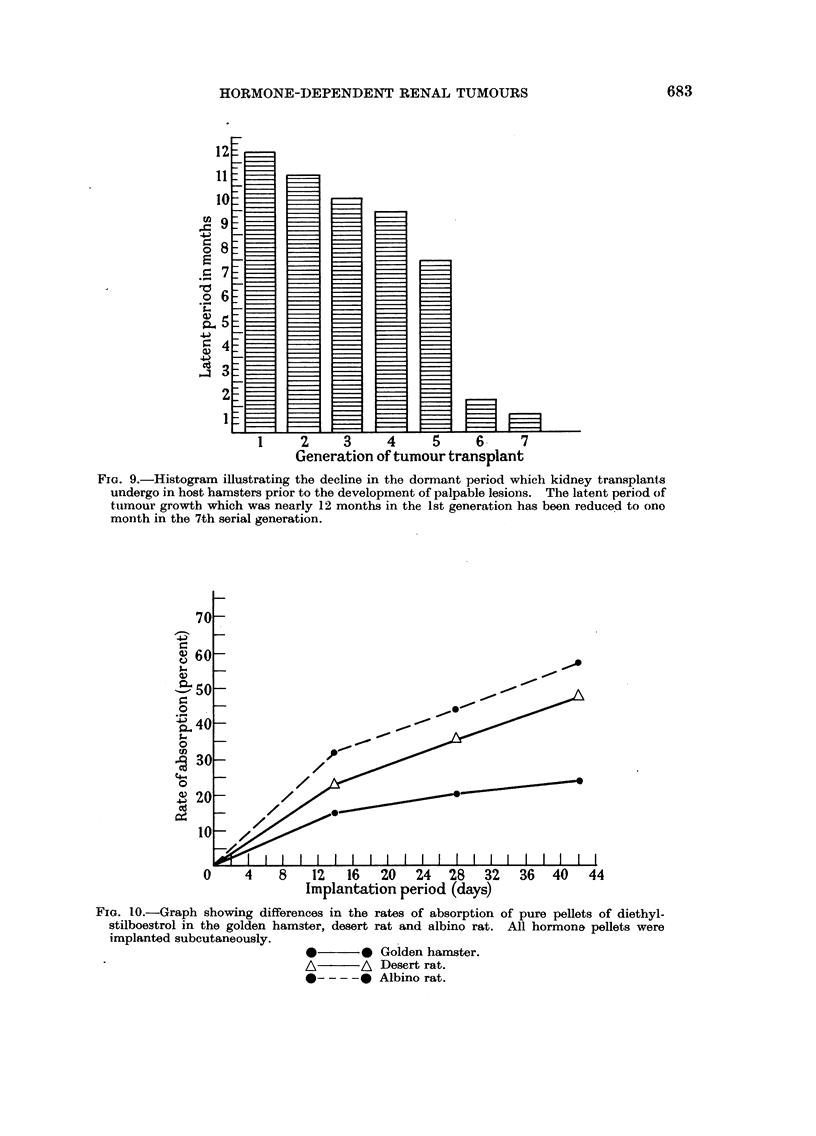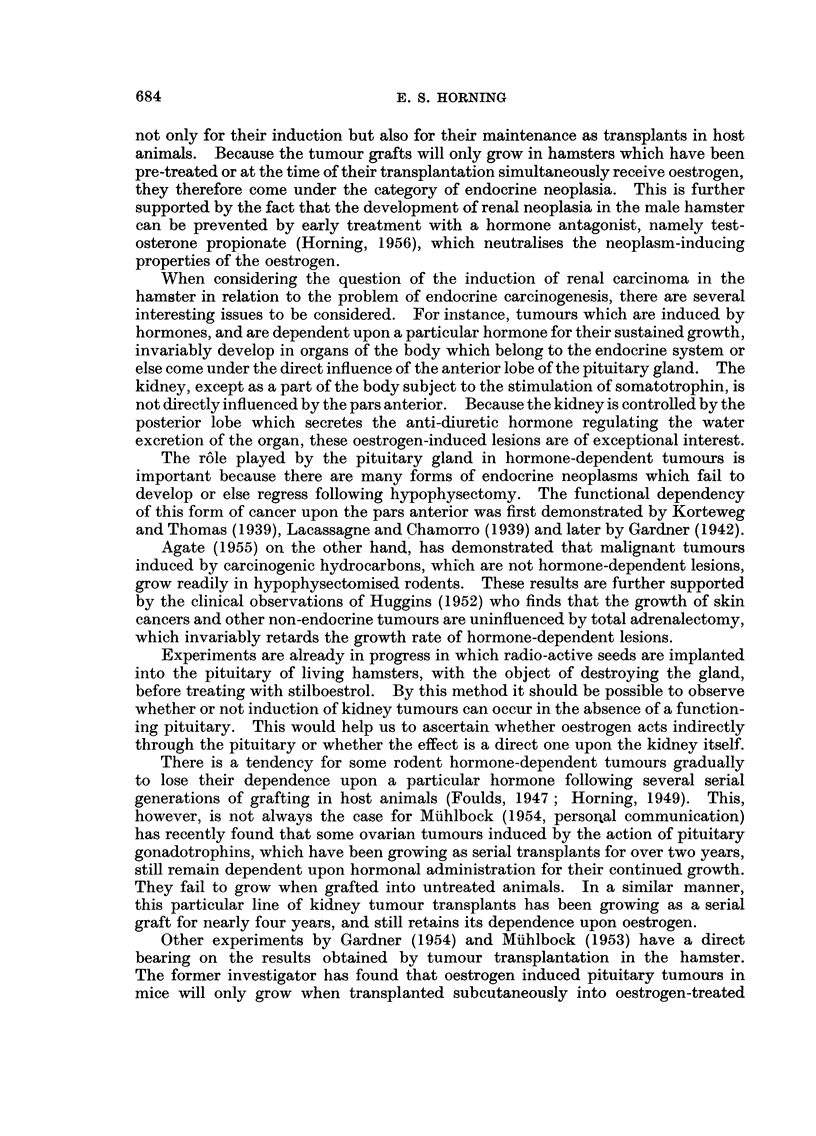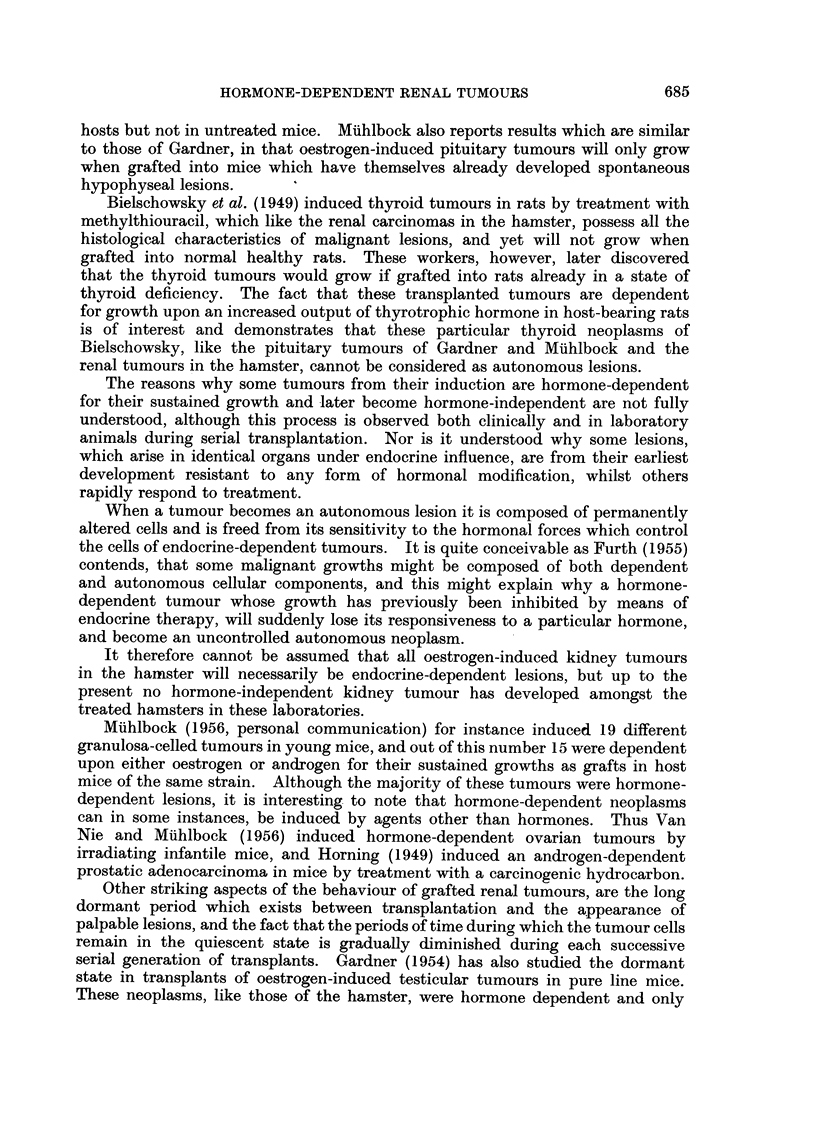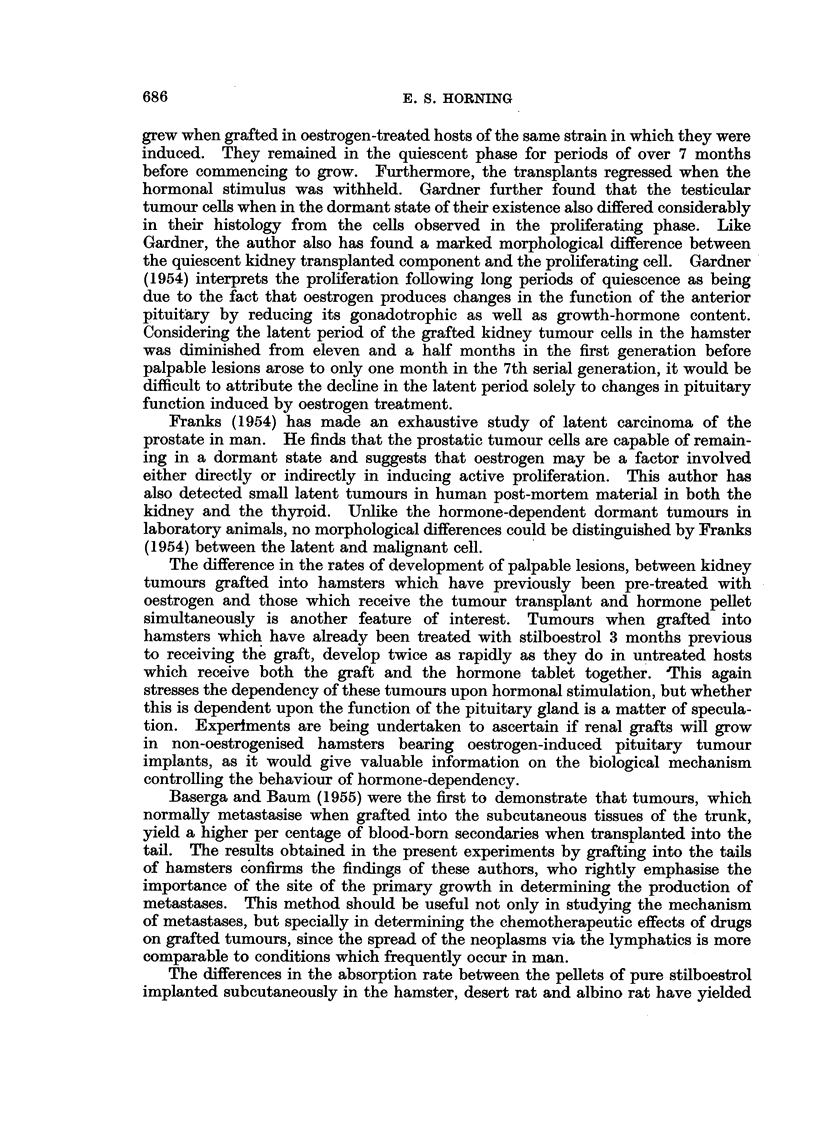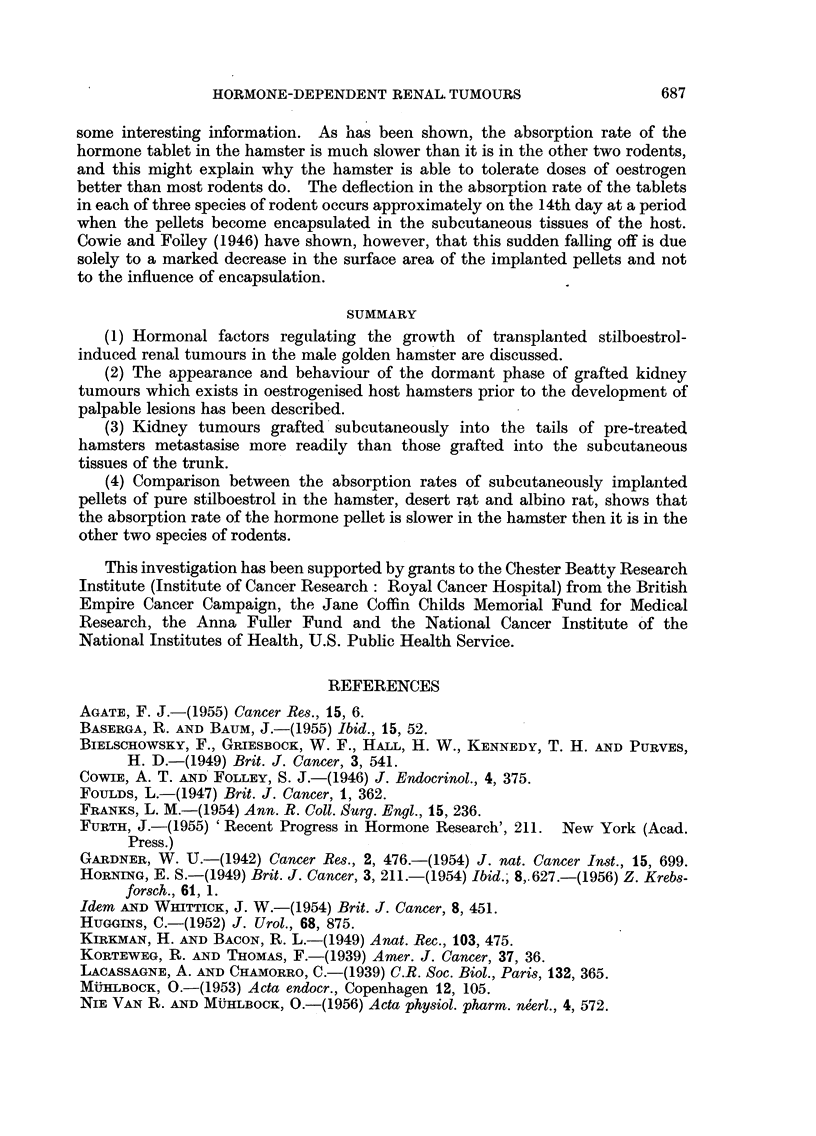# Observations on Hormone-Dependent Renal Tumours in the Golden Hamster

**DOI:** 10.1038/bjc.1956.82

**Published:** 1956-12

**Authors:** E. S. Horning

## Abstract

**Images:**


### OBSERVATIONS ON HORMONE-DEPENDENT RENAL TUMOURS IN THE GOLDEN HAMSTER

E. S. HORNING

*From the Chester Beatty Research Institute, Institute of Cancer Research : Royal Cancer Hospital, Fulham Road, London, S.W.3*

Received for publication August 9, 1956

In a previous paper Horning (1954) reported the hormonal factors determining the successful transplantation of stilboestrol-induced renal tumours in the male golden hamster, the development of which was first described by Kirkman and Bacon (1949). Since then, these experiments on transplantation have been extended to provide additional evidence that renal neoplasia in the hamster comes under the category of hormonal cancer. The results are discussed herein in the light of our present knowledge of endocrine neoplasia in other species of laboratory animals.

### MATERIAL AND METHODS

Male golden hamsters of indeterminate ancestry bred in this Institute were used exclusively in these experiments. Primary kidney tumours were induced by implanting 20 mg. pellets of diethyl-stilboestrol subcutaneously in each animal. Each pellet was made from the pure synthetic hormone. They were moulded under pressure, and no binding medium was used. Each tablet was weighed prior to implantation, and each hamster received a second stilboestrol pellet 12 weeks after the first had been implanted. The period of induction between pellet implantation and the development of palpable kidney tumours was considerably reduced following treatment with the second tablet and the incidence of kidney tumours was also increased. Approximately 70 to 80 per cent of all male hamsters develop palpable renal lesions within 9 to 12 months following treatment with a single 20 mg. pellet, whereas the incidence following the introduction of the second is in some instances increased to 100 per cent. It is preferable, although not absolutely necessary, to select hamsters 6 to 8 weeks for this purpose.

The most satisfactory way of obtaining successful transplantable renal tumours was to graft them subcutaneously into host animals which have been pre-treated with a 20 mg. tablet of stilboestrol 3 months prior to receiving the tumour implant. In another separate series of experiments both the stilboestrol pellet and the tumour were grafted simultaneously into normal hamsters which had not been previously treated with the synthetic hormone. The differences in the growth rates between the tumour grafts in these two series of experiments were then later compared.

Tumours were also grafted into the subcutaneous tissues of the tail as well as into the flanks of pre-treated male hamsters. Selected pieces of the kidney tumour were finely minced up in saline, and approximately 0·2 ml. of this suspension was injected. The tail of the hamster is very small, and must be held firmly on a thick cork mat. The point of the syringe needle is then directed towards the tip of the tail, taking precaution to avoid the caudal vein.

All tumours transplanted into oestrogenised hamsters undergo long latent periods in the subcutaneous tissues of their hosts before the development of palpable lesions. The periods of time these tumours remain in the quiescent phase, before active growth commences, is diminished during each successive serial generation of transplants.

Histological studies were made of some of these dormant grafts from the 3rd generation of serial transplants. With the aid of a binocular microscope, quiescent transplants can, with care, be removed from their subcutaneous sites and transferred to fixative without much damage.

Induced renal primary tumours grow as an ascites in the peritoneum of hamsters providing they are transferred into hosts which have previously been oestrogenised. The ascitic kidney tumours were transplanted in 3 serial generations. Material for the 1st transfer was obtained from a hamster with very large multifocal kidney tumours possessing metastatic implants on the abdominal parietes and viscera. Selected pieces of the primary lesion and the implants were finely minced up with saline solution and 0·2 ml. of this suspension was injected intraperitoneally into hamsters all of which had been treated subcutaneously with a 20 mg. pellet 3 months beforehand. At the same time an equal number of hamsters which had not been pre-treated with the synthetic hormone received similar amounts of the saline tumour emulsion intraperitoneally to form the control group.

After a latent period of 6 to 9 months following transfer, the abdomens of 25 per cent of the treated animals became distended with fluid, and smears of this abdominal fluid were prepared for cytological examination. At post mortem, after all the peritoneal fluid had been drawn off, the numerous tumour implants which had grown on the peritoneum, were fixed for histological study.

A study was also undertaken to ascertain if any differences existed between the absorption rates of the subcutaneously implanted stilboestrol pellets when transplanted under identical conditions in male hamsters, albino and desert rats. Twenty mg. tablets of pure stilboestrol were implanted subcutaneously into the left flanks of the three different species of male rodents, each animal being 12 weeks of age at the time of implantation. The implanted hormone tablets were recovered from the rodents at intervals ranging from 7 to 45 days. They were then dried and weighed, and the absorption over a given period was worked out separately for each species of rodent.

For routine histological study, kidney tumour material was fixed either in aqueous or alcoholic Bouin and subsequently stained in either haematoxylin and eosin or by a modification of Masson's light green stain. Pituitary glands were fixed in Zenker-formol and differentially stained by a modification of Mallory's triple stain.

### *Observations*

### *Endocrine factors involved in successful tumour transplantation*

Since the macroscopic appearance and methods of induction of primary kidney tumours in the hamster by oestrogen have been previously reported by Kirkman and Bacon (1949) and by Horning and Whittick (1954), this communication will be concerned mainly with factors controlling the successful transplantation of the tumours. However, for the sake of convenience the macroscopic appearance of a typical primary multifocal and bilateral renal carcinoma is illustrated in

Fig. 1. The majority of kidney tumours were induced $10\frac{1}{2}$ months following the subcutaneous implantation of a 20 mg. stilboestrol pellet, which was followed by a second similar tablet 3 months after the first had been implanted (Fig. 2).

Many attempts have been made without success to graft these induced primary renal tumours in host hamsters. Tumour transplants failed to grow, however, either when grafted subcutaneously or intraperitoneally into intact untreated hamsters of both sexes and of varying ages (Horning, 1954). In several instances metastatic implants from the diaphragm were also transplanted but in every instance these also failed to grow. The untreated hamsters bearing the tumour grafts were kept alive for a year, and were subsequently killed as no palpable lesions had developed. Many animals with negative grafts were killed at intervals varying from 4 to 12 months after transplantation. In every instance both the tumours grafted subcutaneously and those transferred intraperitoneally had been totally absorbed by the host-bearing animals.

Since these primary kidney carcinomas possessed all the histological characteristics of malignant growths and metastasised in many instances via the lymphatic pathway (Horning, 1956), their failure to grow either as subcutaneous grafts or intraperitoneal transfers was surprising.

Consideration was then given to the fact that as the kidney tumours are dependent upon high levels of estrogen for their induction they might also be dependent upon the continuous stimulation of oestrogen in excessive amounts for their maintenance as transplants in host animals. Selected pieces of a large primary kidney tumour were grafted into male hamsters all of which had been previously treated for 3 months with a 20 mg. pellet of stilboestrol. An equal number of normal untreated males likewise received subcutaneous grafts from the same kidney tumour, and these acted as controls. Small palpable nodules began to develop 7 to 8 months in 45 per cent of the stilboestrol-treated hosts, whilst none developed in the untreated group. The experiments were repeated several times with the same results and conclusively demonstrated that these hamster renal tumours are not autonomus lesions since they are dependent upon oestrogen for both their induction and maintenance as grafts (Fig. 3).

The results of grafting the kidney tumours into the subcutaneous tissues of the tails in pre-treated hamsters (Fig. 8) showed conclusively that a higher percentage of blood-borne metastases developed, specially in the lymph nodes, than normally occurs when tumours are implanted into the trunk.

Experiments are being undertaken on the regression of these transplanted tumours following the withdrawal of the hormone pellet. These observations are easier to determine when growing in the tail than they are in the trunk.

Histological examination of the first generation of tumour transplants growing in pre-treated hosts showed them to be actively growing clear-celled carcinomas (Horning, 1954). Although these kidney tumour grafts are now in their 8th generation of serial grafts they still show no evidence of anaplasia (Fig. 7). Also 95 to 100 per cent of the 8th generation continue to grow provided they are transplanted into pre-treated hosts.

Microscopic examination of the ascitic tumours which had grown on the peritoneum, and surfaces of the kidney and other organs, were also similar in their histology to the primary lesion from which the tumour suspension was made. Ascitic tumour deposits on the diaphragm as well as on the renal capsule are seen in Fig. 4. Marcoscopically the lesions have the appearance of early primary cortical growths, but subsequent histological examination showed them to be only colonies of tumour ascitic cells growing in compact formation and not early primary renal tumours. The tumour implants developed from clumps of ascitic cells which first adhered to the renal capsule and subsequently multiplied, thus forming foci of solid tumours (Fig. 5). Microscopic examination of both kidneys failed to reveal the presence of any primary renal lesions. Another interesting feature was that the peritoneal fluid of these hamsters contained remarkably few ascitic tumour cells. Apart from the kidney tumour cells the fluid contained some detached mesothelial cells and leucocytes. Smears of the peritoneal fluid showed that the ascitic tumour cells were invariably seen in small clumps. Although freed from their complicated stroma and growing in a new medium they were easily recognised. They possessed large nuclei with little chromatin, with a faintly staining cytoplasm, and resembled the clear-celled carcinoma elements which composed the solid tumours (Fig. 6).

When growing as an ascites tumour, the renal carcinomas behave in much the same way as the subcutaneous solid tumours. Following transfer into the peritoneal fluid of pre-treated host animals, they undergo long dormant periods before they begin to multiply and finally enter the malignant phase. But compared with the solid tumours, approximately only 30 per cent of the ascites tumours had grown following transfer.

A study of the histogram (Fig. 9) of subcutaneous transplanted tumours shows that this latent period preceding growth of the graft is gradually reduced following each successive generation of transplants. For instance, in the 1st generation of transplants in one particular line of kidney tumour grafts (Fig. 9) the cells of the transplants remained in the quiescent state for nearly 11½ months before they exhibited any sign of active growth. The 4th generation of renal grafts showed that the dormant period was considerably reduced and that palpable lesions developed 6 to 7 months after grafting, whereas in the 6th generation palpable tumours occurred as early as 2 months following implantation.

One strain of the ascites kidney tumour is now in its 3rd generation of transfer. The 1st generation remained dormant in the peritoneal fluid for nearly 7 months. By this time the abdomens of the host animals became distended with fluid and had to be sacrificed. The 2nd generation took 5 months, and the 3rd 4½ months, before abdominal distention occurred.

Although the ascitic tumour cells in their 1st and 2nd generations of transfer do not remain in the quiescent phase for such long periods of time as the solid kidney grafted tumours, it is of interest to note that in each case the dormant phase of the transplanted cell growing in the peritoneal fluid is likewise gradually reduced during each successive generation.

The histology of the kidney tumour cells growing as solid tumours has been studied during their quiescent phase. Their morphology during the dormant state contrasts greatly when compared with those tumour cells which have entered into the malignant phase. The cells have a dehydrated appearance, are irregular in outline, and both the cytoplasm and the nuclei stain darkly. In many instances it is difficult to differentiate between the shrunken deeply stained nuclei and the cytoplasm. The cells composing a transplant which had suddenly commenced to grow, differed greatly when compared to those forming a dormant graft. The cells were spherical in outline and both the cytoplasm and the nuclei were only faintly stained ; also numerous cells are seen in division.

The structure and behaviour of the ascitic tumour cells during their dormant phase in the peritoneal fluid, owing to obvious technical difficulties, have not yet been studied.

*Comparison of absorption rates of subcutaneously implanted stilboestrol pellets in male hamster, albino and desert rats*

The fact that hamsters can tolerate very large doses of both the naturally-occurring and the synthetic oestrogens is also of interest. Comparative experiments were therefore undertaken to ascertain the differences, if any, between the rates of absorption of subcutaneously transplanted stilboestrol pellets in the male hamster, and in albino and desert rats. The implanted tablets of pure stilboestrol were recovered from each species of rodent at the same time, at intervals ranging from 7 to 45 days. After the pellets were dried and weighed following various intervals of recovery, a considerable difference between the loss of weight in the tablets recovered from each of the species was clearly noticeable. The rate of absorption of the pellets was found to be considerably slower in the hamster than in either the albino rat or the desert rat (Fig. 10).

In the hamster approximately 24 per cent of the pure stilboestrol pellet was absorbed after 42 days implantation, whilst in the desert rat 45 per cent was absorbed and in the albino rat 55 per cent. After the tablets had been subcutaneously implanted for a period of 14 days it was observed that a marked decrease occurred in the absorption rate of the pellets in each of the three different species of rodent (Fig. 10). It was of interest to note that this deflection occurred at a time when each of the implanted pellets had become encapsulated in the subcutaneous tissues of the host animals.

### DISCUSSION

The results of these experiments show conclusively that kidney tumours in the male golden hamster are dependent upon excessive oestrogenic stimulation

---

### EXPLANATION OF PLATES

Fig. 1.—Macroscopic photograph of multifocal and bilateral kidney tumours in a male golden hamster following 10½ months treatment with two 20 mg. pellets of pure stilboestrol. × 1·7.

Fig. 2.—Macroscopic photograph showing a stilboestrol-induced multifocal renal tumour in one kidney, together with two stilboestrol pellets (indicated by arrows) implanted in the subcutaneous tissues of the host. The smaller of the two tablets, the first to be transplanted, had been implanted for six months and the second pellet for three months. × 1·9.

Fig. 3.—Hormone-dependent kidney tumour growing subcutaneously in a male hamster which had been pre-treated with stilboestrol. The two arrows indicate the hormone pellets. × 1.

Fig. 4.—Macroscopic photograph of a male hamster pre-treated with stilboestrol in which the kidney tumour had been growing as an ascites. Note the tumour implants on the diaphragm, as well as on the capsule of the kidney (see arrow). × 1·7. Compare with Fig. 5.

Fig. 5.—Section of the tumour implant indicated by an arrow in Fig. 4. The tumour had been growing as an ascites and formed a colony on the capsule of the kidney. Macroscopically these lesions growing on the surface of the kidney have the appearance of primary tumours. × 720.

Fig. 6.—Smear preparation made from abdominal fluid of a hamster bearing a kidney tumour as an ascites. Observe the cluster of tumour cells. × 720.

Fig. 7.—Section of a generation solid tumour transplant. This tumour is a clear-cell carcinoma and has not undergone anaplastic changes. × 130.

Fig. 8.—Kidney tumour graft growing in tail of a stilboestrol pre-treated male hamster × 1·3.

1

2

3

4

5

6

Horning.

7

8

Horning.

Fig. 9.—Histogram illustrating the decline in the dormant period which kidney transplants undergo in host hamsters prior to the development of palpable lesions. The latent period of tumour growth which was nearly 12 months in the 1st generation has been reduced to one month in the 7th serial generation.

Fig. 10.—Graph showing differences in the rates of absorption of pure pellets of diethylstilboestrol in the golden hamster, desert rat and albino rat. All hormone pellets were implanted subcutaneously.

●——● Golden hamster.
△——△ Desert rat.
●- - - -● Albino rat.

not only for their induction but also for their maintenance as transplants in host animals. Because the tumour grafts will only grow in hamsters which have been pre-treated or at the time of their transplantation simultaneously receive oestrogen, they therefore come under the category of endocrine neoplasia. This is further supported by the fact that the development of renal neoplasia in the male hamster can be prevented by early treatment with a hormone antagonist, namely testosterone propionate (Horning, 1956), which neutralises the neoplasm-inducing properties of the oestrogen.

When considering the question of the induction of renal carcinoma in the hamster in relation to the problem of endocrine carcinogenesis, there are several interesting issues to be considered. For instance, tumours which are induced by hormones, and are dependent upon a particular hormone for their sustained growth, invariably develop in organs of the body which belong to the endocrine system or else come under the direct influence of the anterior lobe of the pituitary gland. The kidney, except as a part of the body subject to the stimulation of somatotrophin, is not directly influenced by the pars anterior. Because the kidney is controlled by the posterior lobe which secretes the anti-diuretic hormone regulating the water excretion of the organ, these oestrogen-induced lesions are of exceptional interest.

The rôle played by the pituitary gland in hormone-dependent tumours is important because there are many forms of endocrine neoplasms which fail to develop or else regress following hypophysectomy. The functional dependency of this form of cancer upon the pars anterior was first demonstrated by Korteweg and Thomas (1939), Lacassagne and Chamorro (1939) and later by Gardner (1942).

Agate (1955) on the other hand, has demonstrated that malignant tumours induced by carcinogenic hydrocarbons, which are not hormone-dependent lesions, grow readily in hypophysectomised rodents. These results are further supported by the clinical observations of Huggins (1952) who finds that the growth of skin cancers and other non-endocrine tumours are uninfluenced by total adrenalectomy, which invariably retards the growth rate of hormone-dependent lesions.

Experiments are already in progress in which radio-active seeds are implanted into the pituitary of living hamsters, with the object of destroying the gland, before treating with stilboestrol. By this method it should be possible to observe whether or not induction of kidney tumours can occur in the absence of a functioning pituitary. This would help us to ascertain whether oestrogen acts indirectly through the pituitary or whether the effect is a direct one upon the kidney itself.

There is a tendency for some rodent hormone-dependent tumours gradually to lose their dependence upon a particular hormone following several serial generations of grafting in host animals (Foulds, 1947 ; Horning, 1949). This, however, is not always the case for Mühlbock (1954, personal communication) has recently found that some ovarian tumours induced by the action of pituitary gonadotrophins, which have been growing as serial transplants for over two years, still remain dependent upon hormonal administration for their continued growth. They fail to grow when grafted into untreated animals. In a similar manner, this particular line of kidney tumour transplants has been growing as a serial graft for nearly four years, and still retains its dependence upon oestrogen.

Other experiments by Gardner (1954) and Mühlbock (1953) have a direct bearing on the results obtained by tumour transplantation in the hamster. The former investigator has found that oestrogen induced pituitary tumours in mice will only grow when transplanted subcutaneously into oestrogen-treated hosts but not in untreated mice. Mühlbock also reports results which are similar to those of Gardner, in that oestrogen-induced pituitary tumours will only grow when grafted into mice which have themselves already developed spontaneous hypophyseal lesions.

Bielschowsky *et al.* (1949) induced thyroid tumours in rats by treatment with methylthiouracil, which like the renal carcinomas in the hamster, possess all the histological characteristics of malignant lesions, and yet will not grow when grafted into normal healthy rats. These workers, however, later discovered that the thyroid tumours would grow if grafted into rats already in a state of thyroid deficiency. The fact that these transplanted tumours are dependent for growth upon an increased output of thyrotrophic hormone in host-bearing rats is of interest and demonstrates that these particular thyroid neoplasms of Bielschowsky, like the pituitary tumours of Gardner and Mühlbock and the renal tumours in the hamster, cannot be considered as autonomous lesions.

The reasons why some tumours from their induction are hormone-dependent for their sustained growth and later become hormone-independent are not fully understood, although this process is observed both clinically and in laboratory animals during serial transplantation. Nor is it understood why some lesions, which arise in identical organs under endocrine influence, are from their earliest development resistant to any form of hormonal modification, whilst others rapidly respond to treatment.

When a tumour becomes an autonomous lesion it is composed of permanently altered cells and is freed from its sensitivity to the hormonal forces which control the cells of endocrine-dependent tumours. It is quite conceivable as Furth (1955) contends, that some malignant growths might be composed of both dependent and autonomous cellular components, and this might explain why a hormone-dependent tumour whose growth has previously been inhibited by means of endocrine therapy, will suddenly lose its responsiveness to a particular hormone, and become an uncontrolled autonomous neoplasm.

It therefore cannot be assumed that all oestrogen-induced kidney tumours in the hamster will necessarily be endocrine-dependent lesions, but up to the present no hormone-independent kidney tumour has developed amongst the treated hamsters in these laboratories.

Mühlbock (1956, personal communication) for instance induced 19 different granulosa-celled tumours in young mice, and out of this number 15 were dependent upon either oestrogen or androgen for their sustained growths as grafts in host mice of the same strain. Although the majority of these tumours were hormone-dependent lesions, it is interesting to note that hormone-dependent neoplasms can in some instances, be induced by agents other than hormones. Thus Van Nie and Mühlbock (1956) induced hormone-dependent ovarian tumours by irradiating infantile mice, and Horning (1949) induced an androgen-dependent prostatic adenocarcinoma in mice by treatment with a carcinogenic hydrocarbon.

Other striking aspects of the behaviour of grafted renal tumours, are the long dormant period which exists between transplantation and the appearance of palpable lesions, and the fact that the periods of time during which the tumour cells remain in the quiescent state is gradually diminished during each successive serial generation of transplants. Gardner (1954) has also studied the dormant state in transplants of oestrogen-induced testicular tumours in pure line mice. These neoplasms, like those of the hamster, were hormone dependent and only grew when grafted in oestrogen-treated hosts of the same strain in which they were induced. They remained in the quiescent phase for periods of over 7 months before commencing to grow. Furthermore, the transplants regressed when the hormonal stimulus was withheld. Gardner further found that the testicular tumour cells when in the dormant state of their existence also differed considerably in their histology from the cells observed in the proliferating phase. Like Gardner, the author also has found a marked morphological difference between the quiescent kidney transplanted component and the proliferating cell. Gardner (1954) interprets the proliferation following long periods of quiescence as being due to the fact that oestrogen produces changes in the function of the anterior pituitary by reducing its gonadotrophic as well as growth-hormone content. Considering the latent period of the grafted kidney tumour cells in the hamster was diminished from eleven and a half months in the first generation before palpable lesions arose to only one month in the 7th serial generation, it would be difficult to attribute the decline in the latent period solely to changes in pituitary function induced by oestrogen treatment.

Franks (1954) has made an exhaustive study of latent carcinoma of the prostate in man. He finds that the prostatic tumour cells are capable of remaining in a dormant state and suggests that oestrogen may be a factor involved either directly or indirectly in inducing active proliferation. This author has also detected small latent tumours in human post-mortem material in both the kidney and the thyroid. Unlike the hormone-dependent dormant tumours in laboratory animals, no morphological differences could be distinguished by Franks (1954) between the latent and malignant cell.

The difference in the rates of development of palpable lesions, between kidney tumours grafted into hamsters which have previously been pre-treated with oestrogen and those which receive the tumour transplant and hormone pellet simultaneously is another feature of interest. Tumours when grafted into hamsters which have already been treated with stilboestrol 3 months previous to receiving the graft, develop twice as rapidly as they do in untreated hosts which receive both the graft and the hormone tablet together. This again stresses the dependency of these tumours upon hormonal stimulation, but whether this is dependent upon the function of the pituitary gland is a matter of speculation. Experiments are being undertaken to ascertain if renal grafts will grow in non-oestrogenised hamsters bearing oestrogen-induced pituitary tumour implants, as it would give valuable information on the biological mechanism controlling the behaviour of hormone-dependency.

Baserga and Baum (1955) were the first to demonstrate that tumours, which normally metastasise when grafted into the subcutaneous tissues of the trunk, yield a higher per centage of blood-born secondaries when transplanted into the tail. The results obtained in the present experiments by grafting into the tails of hamsters confirms the findings of these authors, who rightly emphasise the importance of the site of the primary growth in determining the production of metastases. This method should be useful not only in studying the mechanism of metastases, but specially in determining the chemotherapeutic effects of drugs on grafted tumours, since the spread of the neoplasms via the lymphatics is more comparable to conditions which frequently occur in man.

The differences in the absorption rate between the pellets of pure stilboestrol implanted subcutaneously in the hamster, desert rat and albino rat have yielded some interesting information. As has been shown, the absorption rate of the hormone tablet in the hamster is much slower than it is in the other two rodents, and this might explain why the hamster is able to tolerate doses of oestrogen better than most rodents do. The deflection in the absorption rate of the tablets in each of three species of rodent occurs approximately on the 14th day at a period when the pellets become encapsulated in the subcutaneous tissues of the host. Cowie and Folley (1946) have shown, however, that this sudden falling off is due solely to a marked decrease in the surface area of the implanted pellets and not to the influence of encapsulation.

### SUMMARY

(1) Hormonal factors regulating the growth of transplanted stilboestrol-induced renal tumours in the male golden hamster are discussed.

(2) The appearance and behaviour of the dormant phase of grafted kidney tumours which exists in oestrogenised host hamsters prior to the development of palpable lesions has been described.

(3) Kidney tumours grafted subcutaneously into the tails of pre-treated hamsters metastasise more readily than those grafted into the subcutaneous tissues of the trunk.

(4) Comparison between the absorption rates of subcutaneously implanted pellets of pure stilboestrol in the hamster, desert rat and albino rat, shows that the absorption rate of the hormone pellet is slower in the hamster then it is in the other two species of rodents.

This investigation has been supported by grants to the Chester Beatty Research Institute (Institute of Cancer Research : Royal Cancer Hospital) from the British Empire Cancer Campaign, the Jane Coffin Childs Memorial Fund for Medical Research, the Anna Fuller Fund and the National Cancer Institute of the National Institutes of Health, U.S. Public Health Service.

### REFERENCES

AGATE, F. J.—(1955) *Cancer Res.*, **15**, 6.
BASERGA, R. AND BAUM, J.—(1955) *Ibid.*, **15**, 52.
BIELSCHOWSKY, F., GRIESBOCK, W. F., HALL, H. W., KENNEDY, T. H. AND PURVES, H. D.—(1949) *Brit. J. Cancer*, **3**, 541.
COWIE, A. T. AND FOLLEY, S. J.—(1946) *J. Endocrinol.*, **4**, 375.
FOULDS, L.—(1947) *Brit. J. Cancer*, **1**, 362.
FRANKS, L. M.—(1954) *Ann. R. Coll. Surg. Engl.*, **15**, 236.
FURTH, J.—(1955) 'Recent Progress in Hormone Research', 211. New York (Acad. Press.)
GARDNER, W. U.—(1942) *Cancer Res.*, **2**, 476.—(1954) *J. nat. Cancer Inst.*, **15**, 699.
HORNING, E. S.—(1949) *Brit. J. Cancer*, **3**, 211.—(1954) *Ibid.*, **8**, 627.—(1956) *Z. Krebsforsch.*, **61**, 1.
*Idem* AND WHITTICK, J. W.—(1954) *Brit. J. Cancer*, **8**, 451.
HUGGINS, C.—(1952) *J. Urol.*, **68**, 875.
KIRKMAN, H. AND BACON, R. L.—(1949) *Anat. Rec.*, **103**, 475.
KORTEWEG, R. AND THOMAS, F.—(1939) *Amer. J. Cancer*, **37**, 36.
LACASSAGNE, A. AND CHAMORRO, C.—(1939) *C.R. Soc. Biol., Paris*, **132**, 365.
MÜHLBOCK, O.—(1953) *Acta endocr.*, Copenhagen **12**, 105.
NIE VAN R. AND MÜHLBOCK, O.—(1956) *Acta physiol. pharm. néerl.*, **4**, 572.